# Integrating social services with disease investigation: A randomized trial of COVID-19 high-touch contact tracing

**DOI:** 10.1371/journal.pone.0285752

**Published:** 2023-05-16

**Authors:** Lisa C. Lu, Derek Ouyang, Alexis D’Agostino, Angelica Diaz, Sarah L. Rudman, Daniel E. Ho

**Affiliations:** 1 Regulation, Evaluation, and Governance Lab, Stanford University, Stanford, California, United States of America; 2 County of Santa Clara Public Health Department, San Jose, California, United States of America; Bach Mai Hospital, VIET NAM

## Abstract

COVID-19 exposed and exacerbated health disparities, and a core challenge has been how to adapt pandemic response and public health in light of these disproportionate health burdens. Responding to this challenge, the County of Santa Clara Public Health Department designed a model of “high-touch” contact tracing that integrated social services with disease investigation, providing continued support and resource linkage for clients from structurally vulnerable communities. We report results from a cluster randomized trial of 5,430 cases from February to May 2021 to assess the ability of high-touch contact tracing to aid with isolation and quarantine. Using individual-level data on resource referral and uptake outcomes, we find that the intervention, randomized assignment to the high-touch program, increased the referral rate to social services by 8.4% (95% confidence interval, 0.8%-15.9%) and the uptake rate by 4.9% (-0.2%-10.0%), with the most pronounced increases in referrals and uptake of food assistance. These findings demonstrate that social services can be effectively combined with contact tracing to better promote health equity, demonstrating a novel path for the future of public health.

## Introduction

Structurally vulnerable communities (*e*.*g*., low-income communities and communities of color) have long suffered from disproportionate disease incidence. These disparities have been associated with socioeconomic barriers, ranging from lack of linguistically and culturally appropriate healthcare services to insufficient food and housing access, and are the consequence of decades of systemic racism [1, 2].

COVID-19 has exacerbated these disparities and highlighted the burdens faced by structurally vulnerable communities in adhering to public health measures. In California, Latinx individuals comprised roughly 39% of the population, but 70% of cases in 2020 [3]. Structurally vulnerable communities also face disparate burdens in fully benefiting from traditional disease investigation measures intended to prevent further spread [1]. These communities make up a larger proportion of essential workers and tend to have living conditions which make isolation and quarantine more difficult [3–5]. Testing may be less accessible to essential workers who are less able to leave work, and isolation and quarantine can be challenging for individuals who live in crowded, multigenerational households or lack accommodating sick leave policies. Compounding these factors are language and cultural barriers and mistrust of the government, which are more pronounced in Latinx and immigrant communities and may prevent clients from fully benefiting from governmental and non-governmental support systems [6–8].

In light of these health inequities, many have criticized the applicability and equity of the conventional public health toolkit for pandemic response (*e*.*g*., testing and contact tracing) and argued that a more comprehensive approach is required to address the social barriers faced by structurally vulnerable communities and prevent widening disparities [8–11]. For instance, public health infrastructure could integrate social services to address the nonmedical and social needs that may be as critical to an individual’s health and well-being as strictly medical ones, while addressing the stigma, privacy concerns, cultural differences, and distrust that structurally vulnerable communities experience in health care systems [12, 13]. Without concurrent delivery of social services to help those in need isolate and quarantine, contact tracing efforts may be dampened at best and made impossible at worst [14].

Even when social services are available, existing social science research has shown that willingness to indicate need for and accept social services, or “uptake”, is often low. There is great interest in understanding why public programs like health insurance and food assistance have low uptake rates relative to eligibility and a demand for quantitative research on programs that improve uptake [15]. Existing research has explored how non-financial barriers like administrative burden and information costs contribute to low uptake and suggests that alleviating that burden, whether through shifting it from the individual onto the state or automatic enrollment, can increase uptake [15–17].

Beginning January 6, 2021, the Public Health Department of the County of Santa Clara, California, launched a novel “high-touch” model of contact tracing that was integrated with social services to better support structurally vulnerable communities [18, 19]. Beyond focusing on identifying contacts, chains of transmission, and health conditions, a special corps of high-touch contact tracers explicitly assessed client resource needs, followed up with clients extensively to provide logistical and emotional support, and provided warm handoffs to resource coordinators. This team was linguistically and culturally competent, connected to and supported by a promotores de salud (the Spanish term for “community health workers”) network [20], familiar with the communities they were serving, and trained on the obstacles faced by structurally vulnerable populations (*e*.*g*., evictions, lack of health insurance). Their role also included resource navigation and case management, and they were well-versed in understanding the eligibility and nature of supportive services. In this paper, we document this program and demonstrate its impact from a cluster randomized trial that randomly assigned cases from eligible (structurally vulnerable) ZIP Codes to high-touch contact tracing.

## Materials and methods

### A high-touch model of contact tracing

Starting in October 2020, the County began hiring and training contact tracers to carry out a high-touch model of contact tracing. Table 1 summarizes the model and compares it to standard contact tracing as practiced by the County at the time. Under the high-touch program, tracers focused on monitoring and support, resource linkage, and care coordination for COVID-positive individuals beyond usual practices. While the standard contact tracing procedure typically only requires a single call, high-touch contact tracing enables staff to provide monitoring and support services for as long as 20 days after the initial interview and refer clients to resources to improve their health and well-being based on assessed risk. For example, a “low-risk” client with adequate food, housing, and healthcare would receive a follow-up call 7 days after the initial interview, while a “high-risk” client with little to no support system and inadequate food or housing would receive a follow-up call within 1–3 days and immediate resource referrals. High-touch contact tracers would refer clients to resources through the County’s Isolation and Quarantine Support Program (IQSP), which provides clients in need with resources, such as cash assistance, rental assistance, groceries or cleaning supplies delivery, and motel placements [21]. More details are provided in S1 Appendix.

**Table 1:** Summary of distinctions between standard and high-touch contact tracing.

	Standard	High-touch
**Case assignment**	Standard procedure	Selected from areas with high Social Vulnerability Index and test positivity rate
**Linguistic competency**	State interpretation service	Bilingual contact tracer
**Cultural competency**	Standard training	Recruitment/hiring requirement
**Special training**	Sexual Orientation, Gender Identity, and Expression (SOGIE) and Racial and Health Equity (RHE)	SOGIE, RHE, Trauma-Informed Care, and focused resource training (*e*.*g*., eviction moratoriums, health coverage)
**Resource navigation**	Access to directory of top-rated community resources compiled by high-touch team	Deeper knowledge and understanding of resources through maintaining the community resource directory, vetting resources, and meeting with representatives from resource providers for training
**Team-based care approach**	None	Warm handoff to resources and clinicians
**Monitoring and support**	Single follow-up call	Extended (*i*.*e*., daily calls, follow-up to confirm resource linkage)
**Case management**	None	Assistance with documents, letters, State Disability Insurance applications, referrals, reporting concerns, and complaint portal

The program also borrowed elements from the promotores de salud and community health worker models [22–24]. High-touch contact tracers were linguistically and culturally competent and trained to advocate for individuals and communities facing a variety of obstacles (*e*.*g*., documentation status concerns) and motivate them to obtain care and resources. Their responsibilities subsumed resource navigation and case management, as they received focused resource training; gained a deeper understanding through vetting, curating, and liaising with local resource providers; and assisted clients with documentation and steps for qualification.

### Cluster randomized trial

When the high-touch program first launched in January 2021, the team members non-randomly targeted a subset of “priority” ZIP Codes (see S2 Appendix for list), defined based on high social vulnerability on the CDC’s index (SVI above 50) and elevated case rates [25, 26]. Beginning February 15, 2021, the high-touch team scaled up to 50 members, enabling greater operational capacity to handle a larger volume of cases while still far short of total cases. We thus designed a cluster randomized-controlled trial to measure the effectiveness of the high-touch program from February to May 2021, compared to conventional contact tracing, in operational conditions. Specifically, we define effectiveness as whether the treatment, assignment to the high-touch program, increases the referral rate (whether a contact tracer opened a referral for a case to IQSP services) and the uptake rate (whether the service was delivered). Clustering and case assignment was done by ZIP Code each week, as individual case assignment was not operationally feasible at the time.

All Santa Clara County ZIP Codes were considered for our randomized assignment scheme, but because of capacity constraints, we designed the trial to equitably allocate high-touch contact tracing to a feasible subset of ZIP Codes. Each week, we calculated the COVID-19 positivity rate for each ZIP Code. We also received an estimate of the County’s operational capacity for the coming week, and translated that to an approximate number of ZIP Codes that could be assigned to the high-touch team. We then selected twice that number of top ZIP Codes, ranked by both positivity rate and SVI, to be eligible for randomized assignment. Per County objectives, the original 12 “priority” ZIP Codes were always eligible to be randomized, though in practice these were at the top of SVI and typically eligible anyway through the protocol. Of the total eligible ZIP Codes, ranging from 16 to 53 over the course of the trial, half were then randomly assigned by computer algorithm for prioritization by the high-touch team; the other half were routed through the standard protocol, along with ineligible ZIP Codes. Prioritization meant that for each batch of assignments, high-touch contact tracers prioritized contacting cases from assigned ZIP Codes, but because of fluctuating operational conditions which often deviated from our weekly forecasts, not all cases from these ZIP Codes were subject to high-touch contact tracing. If there was extra capacity, high-touch team members were assigned cases from the eligible-but-not-assigned ZIP Codes. For an illustration of the assignment scheme in an example week, as well other exceptions to the assignment scheme that were of de minimis magnitude, see S3 Appendix.

The Santa Clara County Public Health Department and Stanford University, Stanford, California, deemed this work public health surveillance; the Revised Common Rule deems “public health surveillance activities” not subject to IRB oversight or requirements for informed consent under 45 CFR § 46. Thus, it was not submitted for IRB approval, but was subjected to privacy and compliance review by Santa Clara County.

### Data sources

To analyze the impact of the program on the outcomes of interest, referral and uptake rates, we leveraged two data sources. First, we utilized individual-level data from CalCONNECT, a state-managed system built on a Salesforce cloud platform that the County and other local public health departments in California used to manage and investigate COVID-19 cases. Within CalCONNECT, contact tracers could create referrals to resource coordinators and clinicians as needed for each client. We observe whether a contact tracer opened a referral for a given individual, as well as the identified need: cash assistance, rental assistance, groceries or cleaning supplies, and motel placement. CalCONNECT also contains demographic data (*e*.*g*., gender, race/ethnicity, age) for individuals, allowing us to compare the treatment and control groups along those dimensions in a balance check.

Second, we utilized IQSP data used for tracking resource delivery for individuals. These data track whether resources ended up being approved or delivered to the individual, allowing us to measure uptake rate overall and for the primary IQSP services of rental assistance, monetary reimbursements for food and cleaning supplies, and motel placement. Uptake records on cash assistance were unavailable.

### Analysis

For all outcomes of interest, we estimate intention-to-treat (ITT) effects and complier average causal effects (CACE). For both analyses, we restrict our analysis to jurisdictions participating in the IQSP, as cases elsewhere were routed to their city-specific support programs and not considered for most IQSP services [21]. This leaves us with 5,430 cases between February 15 and May 22, 2021, the time period for participant recruitment into the trial, with all participant follow-up completed by July 21, 2021. We perform a balance check between the treatment and control groups along demographic covariates from CalCONNECT (gender, race/ethnicity, age) to ensure the two groups are comparable.

For the ITT effects, we consider only the assignment status of the cases by the weekly ZIP Code randomization. Since treatment assignment is randomized by week and ZIP Code, we cluster standard errors by week-ZIP Code.

To calculate the CACE, we leverage the weekly ZIP Code randomization as high-touch contact tracing was deployed. Specifically, we estimate the effect among the subgroup of cases that were assigned to the high-touch team solely due to the ZIP Code randomization. This allows us to account for “noncompliance” from randomizing at the ZIP Code level and fluctuating case rates and operational capacity, which led to some deviations from the assignment protocol. We conduct an instrumental variables (IV) analysis, with the instrumental variable being whether the case was assigned to the treatment group via randomization. We include demographic covariates (gender, race/ethnicity, age) as covariates in our regression, specify time (week) fixed effects as controls, and cluster standard errors by week-ZIP Code. More details and robustness checks are provided in S3 Appendix.

### Additional analyses

We perform three additional analyses, which are detailed in the Supporting Information. First, to validate the implementation of the high-touch model, we analyze interview, call, and contact statistics across full-time contact tracers to confirm that high-touch contact tracers indeed had a greater degree of interaction and guidance with clients than standard contact tracers. We perform *t*-tests between high-touch and standard contact tracers on a random sample of call data (see S1 Appendix).

Second, we analyze the “pilot phase” of the high-touch program, which ran from January 6, 2021 until the randomized trial began on February 15, 2021. During this time period, the high-touch team was smaller and was only assigned cases from 12 ZIP Codes, with no randomization. We perform a difference-in-differences analysis, using other ZIP Codes as a control group, and we find substantially identical results of the program impact on referral and uptake rates (see S2 Appendix).

Third, to gather qualitative data on client outcomes and contact tracer experiences from the program, we administered a survey to all high-touch contact tracers active in November 2021. We then identify common themes in the responses (see S4 Appendix).

### Limitations

We note several limitations of our study. First, due to data integration issues, we are unable to observe uptake on all resources available (*e*.*g*., health insurance enrollment, vaccination) or track impacts on isolation ability, disease incidence, and hospitalization. Second, because of operational logistics, the power of the randomized intervention was more limited than originally planned. Fluctuating case counts and capacity meant that full adherence to ZIP Code assignments was not possible, and changes in lab reporting meant sustaining the randomization became infeasible in May 2021, shortening the observation window. We nonetheless detect statistically significant effects, reported below.

## Results

### Cluster randomized trial

For the cluster randomized trial, 5,430 cases were considered, and 2,591 cases were routed to the high-touch team (47.7%). Table 2 provides covariate balance statistics to confirm that the randomization produced comparable treatment and control groups. We assess balance on individual-level gender, race/ethnicity, and age data sourced from CalCONNECT. The “as randomized” treatment and control groups are comparable, with no statistically significant imbalances.

**Table 2:** Balance check on individual-level demographic attributes between treatment group (cases randomly assigned to a high-touch contact tracer) and control group (cases not assigned to a high-touch contact tracer).

		Control	Treatment	95% CI	*p*-value
Sample size (*n*)		2,839	2,591		
Gender	Male	0.53	0.51	[-0.01, 0.04]	0.30
	Female	0.46	0.48	[-0.04, 0.01]	0.33
	Unknown/other	0.01	0.01	[-0.01, 0.00]	0.75
Race/ethnicity	Latinx	0.48	0.49	[-0.04, 0.01]	0.26
	White	0.12	0.12	[-0.01, 0.03]	0.31
	Asian	0.25	0.25	[-0.02, 0.03]	0.73
	Black	0.02	0.02	[-0.01, 0.01]	0.93
	Not reported	0.05	0.04	[-0.01, 0.02]	0.31
	Multiracial/other	0.07	0.07	[-0.02, 0.01]	0.81
Age	Mean age	36.32	36.07	[-0.77, 1.25]	0.64
	0–17	0.18	0.17	[-0.02, 0.02]	0.81
	18–24	0.12	0.13	[-0.03, 0.01]	0.36
	25–44	0.38	0.37	[-0.02, 0.03]	0.55
	45–64	0.25	0.25	[-0.03, 0.02]	0.63
	65+	0.07	0.07	[-0.01, 0.02]	0.61

Author’s analysis of CalCONNECT data.

**Fig 1:**
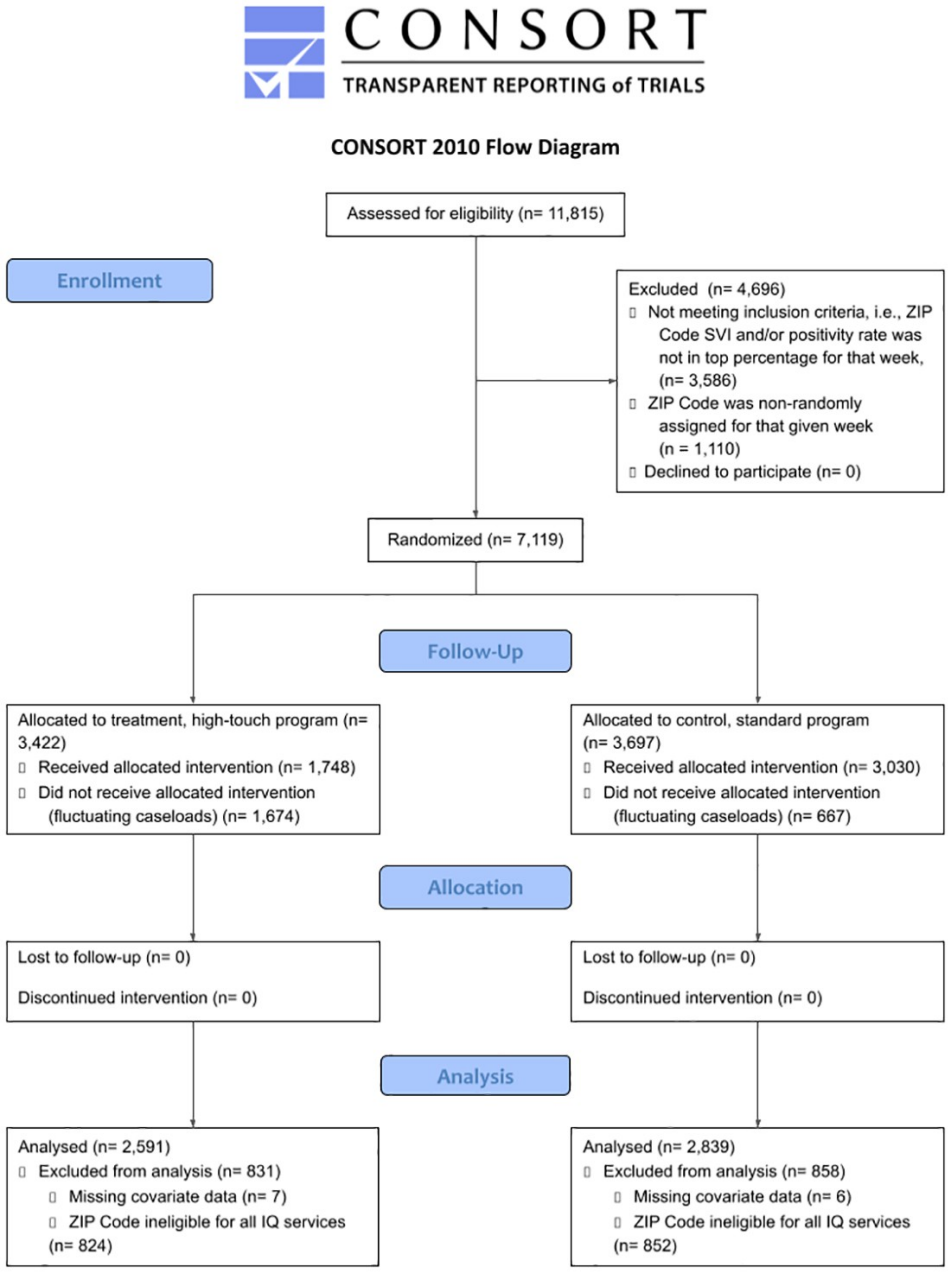
Participant flow diagram following the CONSORT randomized trial reporting recommendations. Author’s analysis of CalCONNECT and Isolation & Quarantine Support Program data. The diagram illustrates the number of clients at each stage of the randomized trial.

The full CONSORT participant flow is illustrated in Fig 1. Of the 2,591 cases routed to the high-touch team, 1,298 (50.1%) were actually assigned to a high-touch contact tracer and the rest to a standard contact tracer due to capacity. Of the 2,839 cases routed to the normal contact tracing process, 2,296 (80.9%) were actually assigned to a standard contact tracer and the rest to a high-touch contact tracer, again because of fluctuating caseloads. These rates of noncompliance reflect the challenge of forecasting both human resources and disease dynamics, as well as the imprecision of the randomization scheme available, but do not equate to any underutilization of available high-touch resources; nor do they present a barrier to our analysis, given our pairing of ITT effects with CACE to expressly account for noncompliance.

We report the results of both analyses in Table 3. The ITT estimates of the high-touch program’s effects on referral and uptake rates are reported on the left-hand side. The F-statistic is 119.33 (*p* < 0.01), confirming that the ZIP Code randomization is a strong instrument. We observe statistically significant increases in the overall referral rate, food assistance referral and uptake, and cleaning supplies uptake. We also see weakly significant evidence of improved overall uptake and cash assistance referral.

**Table 3:** Effect of the high-touch program on referral rates and uptake rates related to Isolation & Quarantine Support Program (IQSP) services as estimated by an intention-to-treat (ITT) analysis on the left-hand side and an instrumental variables (IV) analysis on the right-hand side.

		Intention-to-treat				Instrumental variables			
		Effect	SE	95% CI	*p*-value	Effect	SE	95% CI	*p*-value
**Referral rate (%)**	Overall	2.92**	1.34	[0.30, 5.53]	0.03	8.38**	3.82	[0.82, 15.94]	0.03
	Food assistance	2.22**	0.93	[0.39, 4.04]	0.02	6.61**	2.57	[1.52, 11.71]	0.01
	Cash assistance	1.50*	0.79	[-0.05, 3.06]	0.06	4.07*	2.34	[-0.56, 8.70]	0.08
	Cleaning supplies	0.56	0.71	[-0.82, 1.95]	0.43	1.55	2.09	[-2.59, 5.70]	0.46
	Motel placement	-0.45	0.34	[-1.12, 0.22]	0.19	-1.52	1.12	[-3.74, 0.71]	0.18
	Rental assistance	-0.60	0.63	[-1.82, 0.63]	0.34	-2.22	1.85	[-5.98, 1.46]	0.24
**Uptake rate (%)**	Overall	1.70*	0.90	[-0.07, 3.47]	0.06	4.86*	2.58	[-0.24, 9.97]	0.06
	Food assistance	1.63**	0.75	[0.16, 3.09]	0.03	4.90**	2.13	[0.69, 9.12]	0.02
	Cleaning supplies	1.56**	0.76	[0.07, 3.06]	0.04	4.72**	2.15	[0.46, 8.98]	0.03
	Rental assistance	0.65	0.75	[-0.81, 2.12]	0.38	1.71	2.24	[-2.72, 6.14]	0.45
	Motel placement	0.00	0.14	[-0.28, 0.28]	0.98	-0.01	0.46	[-0.92, 0.90]	0.98

Author’s analysis of CalCONNECT and IQSP data. Effects, standard errors, and 95% confidence interval values are reported as percentages. The data for both these evaluations consists of 5,430 cases. SE = standard error.

**p < 0.05

*p < 0.10.

The IV results are on the right-hand side and largely corroborate the ITT results. Assignment to the high-touch program resulted in an 8.38% (0.82%-15.94%, 95% confidence interval) increase in the overall referral rate, improved food assistance referral and uptake, and improved cleaning supplies uptake, statistically significant at the 5% level. We also see evidence of a 4.86% (-0.24%-9.97%) increase in the overall uptake rate and improved cash assistance referral, though these estimates are statistically significant only at the 10% level. As with the ITT results, we do not observe substantial effects on motel placement or rental assistance referral or uptake rate.

### Additional analyses

The analysis of the high-touch program implementation (detailed in S1 Appendix) shows that high-touch contact tracers indeed offer a greater degree of interaction and guidance than standard contact tracers. High-touch tracers made nearly 3 more call attempts per case, spent 12.52 more minutes making calls per case, and yielded 1.59 more successful follow-up calls post-interview.

The difference-in-differences analysis of the high-touch pilot phase corroborates the randomized results (detailed in S2 Appendix). We observe statistically significant increases in the overall referral rate. The results also show increases in cash assistance, food assistance, rental assistance, and cleaning supplies referral rates, as well as increases in rental assistance, food assistance, and cleaning supplies uptake rates. Consistent with the other analyses, there is no evidence that the program affected the motel placement referral or uptake rate.

Last, the survey responses (summarized in S4 Appendix) indicated the following themes as qualitative benefits to high-touch contact tracing: increased client knowledge about resources; improved physical health outcomes for clients with comorbidities; improved mental well-being for clients; and improved contact tracer morale. The latter is worth noting as contact tracers are particularly at risk for anxiety, stress, and burnout given the demands of their job [27], and may be more generally relevant for retention of frontline workers and the public health workforce [28, 29].

## Discussion

Our study has shown that the high-touch model can improve resource linkage through increased referral and uptake rates for structurally vulnerable communities most impacted by COVID-19. Many have critiqued conventional contact tracing as failing the most structurally vulnerable communities that may be less able to weather the economic impact of a positive diagnosis or the work disruption associated with isolation and quarantine [8]. Our evaluation demonstrates how the high-touch model can improve care for those communities by targeting the highest efficacy services towards those least likely to be able to access supportive services. This is demonstrated not only in the improvements in resource linkage, which showed the greatest gains in areas of food assistance and cleaning supplies, but also in the survey responses, which indicate the model’s potential for alleviating the mental burden faced by clients, improving community knowledge of and trust in the resources available, and bringing contact tracers themselves a greater sense of fulfillment. While our trial exhibited a high rate of noncompliance, where case surges often exceeded the high-touch team’s capacities, the model demonstrated that supportive services, at whatever scale of investment is available, can be feasibly integrated into operations and equitably targeted towards structurally vulnerable communities with measurable benefits. As the public health field strengthens its attention to the social determinants of disease [14, 30–32], the high-touch model serves as one example of integrating community health work into “the full range of health care delivery and population health programs” to make them more accessible [23].

Our findings have several other implications. First, our research-practice partnership demonstrates the feasibility and promise of rigorous program evaluation while in the throes of a public health crisis. The design of a cluster randomized trial, which could be implemented in real-time with the technology available and account for multiple practical assignment and treatment constraints, was possible through a commitment to the principles of co-production, including: sharing of data and operational access; investment of researcher and practitioner time into frequent and reciprocal consultation; and clear translation of technical concepts between academic and public health domains [33, 34].

Second, the high-touch model and its results have implications beyond COVID-19 response. As noted, increasing uptake of public programs has been a sore point in service delivery and an active area of research. The integration of social services and public health may prove useful in increasing uptake in many other settings, such as connecting monkeypox patients with health care resources [35], linking compliance inspections with assistance [36, 37], and streamlining food assistance application processes [38, 39].

Third, our evaluation supports prior research that highlights the substantial role that administrative or logistical complexity may play in low uptake rates of social services. For instance, while the program’s effects were most pronounced in areas of food assistance and cleaning supplies, there was no statistically significant effect on motel placement referral or uptake rate. This is consistent with the simpler eligibility requirements for motel placement and the more complex process for getting approval for food assistance and cleaning supplies, suggesting that the high-touch contact tracers, whose roles included resource navigation, most benefited clients seeking resources with more complex application processes [15, 16]. Another possibility is that client households prefer isolating and quarantining at home over a motel placement which may present logistical issues related to childcare, work, and other lifestyle needs; the efficacy of food assistance and cleaning supplies may reflect this preference for social services that cater to a client’s existing living situation with minimal disruption. In the case of rental assistance, the additional difference-in-difference analysis suggested small but significant effects, even if results were statistically insignificant in the randomized design. Given the ineligibility of homeowners and the existence of a tenant eviction moratorium during the study period, the effect on rental assistance may have been attenuated. This type of evaluation for future programs can inform public health investments in identifying the most appropriate social services for structurally vulnerable communities and improving resource navigation for those that involve complex processes, or simplifying processes altogether.

Last, our results show that community health work can be integrated even in large-scale, urgent public health interventions or areas lacking in existing participation by community health organizations [32, 40]. While the need for staffing and managing a large contact tracing operation led the County to contract with a non-local organization, they required hiring to be local and worked with local community health partners to design job descriptions and recruit, ensuring that contact tracers represented the community. Ultimately, at a time when many jurisdictions scaled down contact tracing operations, based on the results of our trial, the County decided to retain its high-touch model for months longer than the rest of their contact tracing program, to continue aiding the most structurally vulnerable clients, and to ultimately sustain social support elements of the model even when the contact tracing components were retired [41]. The high-touch model demonstrates how contact tracing can be deployed to meet health equity objectives by integrating disease control and outbreak investigation with resource linkage, two historically distinct divisions. Though a full cost-effectiveness analysis is out of scope here, jurisdictions can weigh the location-specific tradeoff posed by high-touch contact tracing in terms of the number of clients reached versus reducing barriers to isolation and quarantine for structurally vulnerable communities.

## Conclusion

COVID-19 has exposed many ways in which public health and pandemic response must grapple with social determinants of health. This study introduces a new model for contact tracing that integrates social services, rolled out as a cluster randomized trial during the pandemic. The results demonstrate how a high-touch contact tracing model could connect structurally vulnerable populations with much needed services to isolate and quarantine.

## Supporting information

S1 Appendix Program implementation. (DOCX)

S2 Appendix Pilot. (DOCX)

S3 Appendix Cluster randomized trial. (DOCX)

S4 Appendix Survey. (DOCX)
