# Supplementary material for: Integrating social services with disease investigation: A randomized trial of COVID-19 high-touch contact tracing
Source: PLoS One. 2023 May 16;18(5):e0285752. doi: 10.1371/journal.pone.0285752 (PMC10187910; doi:10.1371/journal.pone.0285752)
Supplement: S1 Appendix — (DOCX) [file pone.0285752.s001.docx]

# S1 Appendix. Program Implementation.

As a part of the high-touch program, high-touch contact tracers were instructed to follow-up with clients through monitoring and support calls depending on their assessed risk level after the initial interview. Risk level was often reassessed based on follow-up calls. Table 1 illustrates the factors utilized by team members to assess a client’s risk level and the subsequent number of days until follow-up for each category, along with an example. Monitoring and support continue for up to 20 days (more, if the client’s symptoms and circumstances warrant it), as staff provide resource referrals and ensure that services are provided. High-touch contact tracers were also instructed to invest more time in outreach to clients; for example, team members would make 6 call attempts over 3 days to reach a client compared to standard contact tracers, who were instructed to make 2 call attempts over 1 day before closing a case.

**Table 1. Summary of risk assessment protocol for the high-touch program.**

| **Risk level** | **Considerations** | **Time until follow-up** | **Example** |
| --- | --- | --- | --- |
| No high-touch services | - Declines services, follow-up, and/or investigation | None | Client declines follow-up when asked by tracer. Thus, **no high-touch services** are provided. |
| Low risk | - High capacity to follow up with guidelines and referrals - Articulated clear understanding of guidelines - Is connected with primary care provider (PCP) and/or health system - Low risk due to age and/or comorbidities - Lives with family or close friends - Able to provide for own food and housing support - Mild to moderate symptoms or asymptomatic - Has adequate housing | 7 days | Client reports having moderate symptoms and is currently isolating in their own home. Their family is in quarantine and providing food for them via delivery. Client has a PCP and has left a message for them regarding their COVID-19 diagnosis. The client is **low risk** because they are already in isolation and have access to all resources and a PCP. |
| Medium risk | - Mild to moderate symptoms - Some risk due to age and/or comorbidities - At least 2 days of food and other necessary supplies - Has adequate housing - Is connected to a PCP and/or health system - Has a support system | 3-5 days | Client reports having moderate symptoms and is currently isolating in their own home. Client reports concern for food and financial support but is okay for the next few days. Client reports they have diabetes and is currently in remission for prostate cancer. The client is **medium risk** due to presence of comorbidities, moderate symptoms, and some concern for food access. |
| High risk | - Moderate to severe symptoms - Moderate or high risk due to age and/or comorbidities - Isolated or minimal support system - Has less than 2 days of food - Housing needs for safe isolation/quarantine | 1-3 days | Client reports having moderate symptoms. Client has diabetes and a PCP. Client rents a room and is uncomfortable communicating with their housemates for assistance. Client reports not having any groceries for tomorrow. The client is **high risk** due to presence of comorbidities, minimal food, moderate symptoms, and minimal support system. |

During the initial interview with the client, the tracer screens the client, determining their risk level based on factors listed under “Considerations”, and informs them of the program and pending follow-up. Depending on the risk level, the tracer will check in on the client after the amount of time in “Time until follow-up”. The rightmost column illustrates how the protocol is applied to triage example clients.

Santa Clara County's Isolation and Quarantine Support Program (IQSP) provided the following resources: emergency cash assistance of $2,000 or $2,500, to supplement lost or diminished household income due to isolation or quarantine, restricted to families based on income of 80% area median income or less; one month of rent and utility bills paid to landlords and utility providers, not to exceed $5,000, also restricted to income of 80% AMI; groceries and/or cleaning/hygiene supplies to support persons who can isolate and quarantine at home, limited to one delivery per residence; and motel placements with supportive services for those who cannot safely isolate or quarantine at home or who do not have a home.

To illustrate the implementation of the program, we analyzed interview, call, and contact statistics. Table 2 lists these descriptive results. We consider cases assigned to full-time contact tracers over the evaluation period, January 6, 2021 to May 22, 2021. We consider a full-time contact tracer one who works at least 5 days of the week and has at least one assigned case over the evaluation period. We also filter to tracers who were active as of the end of the evaluation period. These criteria omit volunteers and state-affiliated contact tracers who worked in a more temporary capacity compared to the rest of the contact tracers. This yields 25 standard contact tracers, 39 high-touch contact tracers, 2,497 cases routed to standard contact tracing, and 3,891 cases routed to high-touch contact tracing.

**Table 2. Descriptive statistics of standard and high-touch contact tracing.**

|  |  | Standard | High-touch | $p$-value |
| --- | --- | --- | --- | --- |
| **Language** | English | 0.64 (0.63-0.65) | 0.61 (0.59-0.62) | <0.01 |
|  | Non-English | 0.21 (0.20-0.22) | 0.28 (0.27-0.30) | <0.01 |
|  | Unknown/other | 0.15 (0.14-0.16) | 0.11 (0.10-0.12) | <0.01 |
| **Initial interview outcome** | Completed | 0.75 (0.75-0.76) | 0.79 (0.78-0.81) | <0.01 |
|  | Partially completed | 0.01 (0.01-0.02) | 0.02 (0.01-0.02) | <0.01 |
|  | Couldn’t be reached | 0.20 (0.20-0.21) | 0.15 (0.14-0.16) | <0.01 |
|  | Refused to interview | 0.02 (0.02-0.02) | 0.04 (0.03-0.04) | <0.01 |
|  | No attempt made/missing data | 0.01 (0.01-0.01) | 0.00 (0.00-0.00) | <0.01 |
| **Call statistics** | Number of call attempts | 2.18 | 5.10 | <0.01 |
|  | Total duration per case (m) | 12.51 | 25.04 | <0.01 |
|  | Number of follow-up calls | 0.19 | 1.79 | <0.01 |
| **Contacts** | Number of contacts elicited | 0.20 | 0.49 | <0.01 |

Author’s analysis of CalCONNECT data. Statistics related to language and initial interview outcome are reported as a proportion with the 95% confidence interval in parentheses, with p-values calculated from chi-squared tests. Statistics related to calls and contacts are reported as means for each group, based on t-tests. Language, initial interview outcome, and contacts were assessed over 6,388 cases handled by full-time contact tracers over the evaluation period. Call statistics were calculated over a random sample of 200 cases (100 from each group) from those 6,388 cases.

We use all 6,388 cases to compare language, initial interview outcome, and number of contacts elicited between the two groups. From Table 2 we observe slight but statistically significant differences in the interview characteristics, with the high-touch team members interviewing a greater proportion of clients in non-English languages and having a slightly higher interview completion rate. We also observe that high-touch contact tracers elicited a greater number of contacts from interviews.

For call statistics, we utilize call log data from CalCONNECT. Though the logging was automated, it frequently failed to log whether a call happened and its duration, and calls involving an interpreter were also not logged. Due to this missingness, we could not leverage the entire dataset and, instead, we took a random sample of the 6,388 cases and recorded the number of call attempts (including successful follow-up calls) and total call duration for 100 cases routed to the high-touch team and 100 cases routed to other contact tracing staff. For each case record, we went through all of the notes recorded by the assigned contact tracer to understand the date, time, purpose (*i.e.*, interview or monitoring and support), and outcome of each call attempt. We then used the call log to record the duration of each call attempt, but due to the significant missingness mentioned above, the values presented for total duration per case and number of call attempts should be considered lower bounds on the true amount of time and quantity of calls spent on outreach by the contact tracers. Cross-referencing the notes and the call log allowed us to capture unsuccessful call attempts that were not documented and to handle missing or duplicated calls in the call log. We only include interactions with contact tracers (so we omit calls with other parties like resource coordinators or clinicians in this analysis). After this procedure, we observe that cases assigned to high-touch team members had at least twice as many call attempts as those assigned to standard contact tracers and had at least 12 minutes more time spent on outreach. The distribution of call attempts is illustrated in Fig 1, corroborating the greater number of follow-up calls in the high-touch protocol. We also observe that cases assigned to high-touch team members had more successful follow-up calls, which we consider to be the number of successful calls after an initial interview has been conducted.

**Fig 1.** **Distribution of number of call attempts across a random sample of 200 cases.**


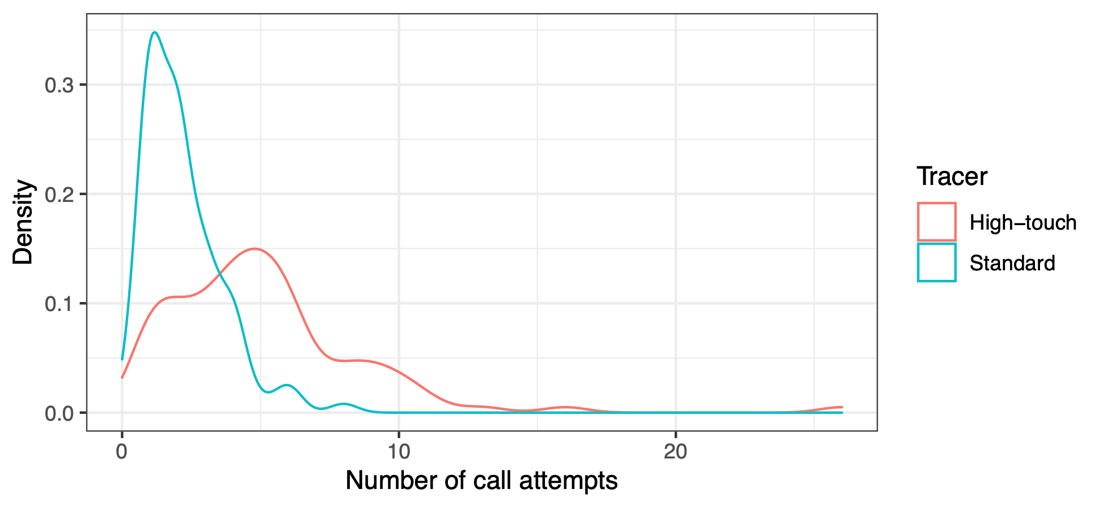


Author’s analysis of CalCONNECT data. The red line represents the distribution for 100 cases assigned to a member of the high-touch team and the blue line represents the distribution for 100 cases assigned to a standard contact tracer.

Operationally, the annual cost for hiring the 50 high-touch contact tracers was approximately $3.2 million. The high-touch contact tracers were a subset of approximately 1,000 contact tracers hired through a $12 million contract with a public health workforce contractor and received the same salary as the standard contact tracers hired through the same organization. High-touch contact tracers received an additional 15 hours of training relative to the standard contact tracers and participated in drop-in, hour-long weekly training and reflective practice sessions throughout the duration of the program.
