# Supplementary material for: Integrating social services with disease investigation: A randomized trial of COVID-19 high-touch contact tracing
Source: PLoS One. 2023 May 16;18(5):e0285752. doi: 10.1371/journal.pone.0285752 (PMC10187910; doi:10.1371/journal.pone.0285752)
Supplement: S2 Appendix — (DOCX) [file pone.0285752.s002.docx]

## S2 Appendix. Pilot.

From January 6, 2021 to February 14, 2021, the County launched a pilot phase of the high-touch program with 36 team members. Cases from 12 “priority” ZIP Codes in East and South San Jose and South County (95116, 95122, 95127, 95111, 95112, 95121, 95148, 95020, 95037, 95123, 95136, and 95128) were routed to the high-touch contact tracing team, while cases from the remaining 46 ZIP Codes in Santa Clara County went through the standard contact tracing protocol. These regions were prioritized due to high social vulnerability (SVI above 50) on the CDC index and elevated case rates [1,2]. Prioritization was done at the ZIP Code level as it was most operationally feasible.

Motivating the differences-in-differences (DiD) analysis of this pilot phase was the observation that the greatest gains in referral and uptake rates from baseline were in a subset of the ZIP Codes that were always assigned to the high-touch team. Table 1 illustrates this: the East San Jose ZIP Codes, a particularly structurally vulnerable area of the County, saw larger increases than the County as a whole in nearly all referral and uptake outcomes, with the exception being motel placement. We hypothesize the low interest in that particular resource to stem from logistical barriers like the client being unable to secure childcare that could not be addressed by the high-touch model. As a result, we decided to leverage data prior to the randomized trial as a way to further assess the impact of the high-touch program on our outcomes of interest.

**Table 1. Comparison of referral and uptake outcomes before (at baseline) and after the high-touch program launch.**

|  |  | **All ZIP Codes** | | | | **East San Jose ZIP Codes** | | | |
| --- | --- | --- | --- | --- | --- | --- | --- | --- | --- |
|  |  | Before | After | 95% CI | $p$-value | Before | After | 95% CI | $p$-value |
| **Referral rate (%)** | Overall | 9.06 | 18.38 | [8.55, 10.07] | <0.01 | 12.92 | 27.50 | [11.81, 17.35] | <0.01 |
|  | Cash assistance | 1.72 | 6.51 | [4.32, 5.27] | <0.01 | 2.79 | 11.92 | [ 7.14, 11.12] | <0.01 |
|  | Rental assistance | 2.52 | 3.23 | [0.37, 1.07] | <0.01 | 4.11 | 5.58 | [ 0.04, 2.90] | 0.04 |
|  | Food assistance | 3.53 | 7.63 | [3.58, 4.62] | <0.01 | 5.77 | 14.52 | [ 6.57, 10.92] | <0.01 |
|  | Cleaning supplies | 0.49 | 3.53 | [2.69, 3.40] | <0.01 | 0.79 | 9.04 | [ 6.49, 10.00] | <0.01 |
|  | Motel placement | 1.02 | 1.28 | [0.04, 0.49] | 0.02 | 1.44 | 1.64 | [-0.60, 0.99] | 0.63 |
| **Uptake rate (%)** | Rental assistance | 2.44 | 3.92 | [1.11, 1.87] | <0.01 | 4.46 | 8.56 | [ 2.37, 5.83] | <0.01 |
|  | Food assistance | 1.99 | 4.79 | [2.39, 3.22] | <0.01 | 3.78 | 10.58 | [ 4.90, 8.70] | <0.01 |
|  | Cleaning supplies | 1.74 | 4.66 | [2.51, 3.33] | <0.01 | 3.35 | 10.48 | [ 5.24, 9.01] | <0.01 |
|  | Motel placement | 0.10 | 0.26 | [0.06, 0.26] | 0.02 | 0.13 | 0.48 | [-0.07, 0.78] | 0.10 |

Author’s analysis of CalCONNECT and IQSP data. We take the baseline period to be the 15-week period before the randomized trial, November 9, 2020 to February 14, 2021.

The way contact tracing was conducted at the beginning of the pandemic was variable, especially as the County was dynamically adjusting to different needs in the community during the case surge in the summer of 2020. It was important to define an observation period during which the differences between the priority ZIP Codes and the rest of the County were stable.

Specifically, in August 2020, the County launched a new mass testing site, the highest capacity test center in the region at the time, to meet testing needs for more structurally vulnerable community members in the priority ZIP Codes [3]. The County also launched pop-up, no-appointment testing sites in the priority ZIP Codes that month [4]. These changes increased testing access to communities in those areas and, through greater testing, increased the number of residents from priority ZIP Codes entering the contact tracing pipeline. As a result, we constrained the observation period to begin after these changes.

For DiD analysis, we set the pre-period as November 7, 2020 to January 5, 2021, before the high-touch program launched, and the post-period as January 6 to February 21, 2021. Our data consists of 79,123 cases, and we compare the change in referral rate and uptake rate for resources between these two periods for the treatment group, the 12 priority ZIP Codes, and the control group, the remaining 46 ZIP Codes. We use a fixed effects model which regresses the outcome on the interaction between treatment time (dummy variable indicating pre-treatment or post-treatment) and treatment ZIP Code (dummy variable indicating whether the ZIP Code was assigned to the high-touch program or not). We account for clustering within ZIP Code and time through two-way cluster-robust standard errors.

Fig 1 presents one of the outcomes, overall referral rate, for the two periods. The referral rate for the treatment group, cases from ZIP Codes that were non-randomly assigned to the high-touch team, is depicted in red, with the referral rate for the control group, cases non-randomly assigned to standard contact tracing teams, shown in blue. The size of the dots represents the case count from CalCONNECT. The vertical line in the plot denotes January 6, 2021, when the pilot began. The figure illustrates that the referral rates for both the treatment and control groups have parallel trends prior to the pilot.

**Fig 1. Referral rate over time for COVID-19 cases prior to randomization.**


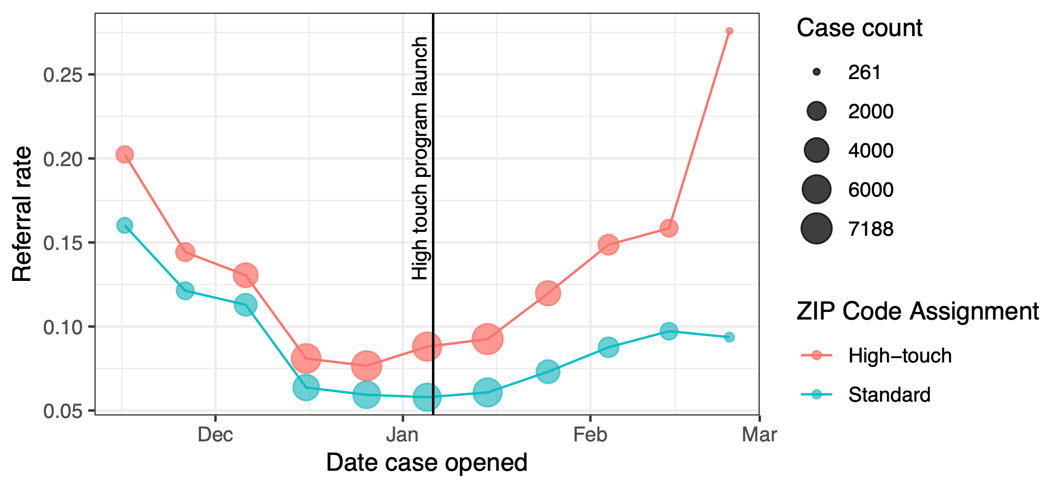


Author’s analysis of CalCONNECT data. The dots represent the mean for the preceding 10-day period, with the size of the dot corresponding to the number of cases. The vertical line denotes January 6, 2021 when the high-touch program pilot officially launched. The dots and line in red represent the referral rate for cases in ZIP Codes non-randomly assigned to the high-touch team beginning January 6. From January 6 to February 14 these were based on the County’s 12 priority ZIP Codes, and during the week of February 15 these were the 3 East San Jose ZIP Codes. The referral rate for cases from the remainder of the ZIP Codes non-randomly assigned to standard contact tracers is depicted in blue. Cases without ZIP Codes or cases subject to randomization were filtered out.

The intervention occurred during the third (Delta) wave of the pandemic, with case counts increasing at the end of 2020. In response to the case surge, the contact tracers switched into “triage mode”, in which only cases were interviewed, contacts received a virtual survey instead of a call, and fewer call attempts were made per case. Though this did not result in fewer referrals, the overall referral rate decreased as the number of cases never reached increased. At the height of the surge, this affected all ZIP Codes, but high-touch contact tracing scaled up as case counts came down in late January and February 2021. We observe that as the high-touch program launched, the referral rates increased dramatically in the pilot ZIP Codes.

Table 2 provides strong evidence that the high-touch program improved referral rates. The results show a 4.18% (1.00%-7.37%) increase in the overall referral rate and a 4.00% (2.26%-5.75%) increase in the cash assistance, a 2.78% (0.63%-4.92%) increase in the food assistance, and a 2.01% (0.77%-3.25%) increase in the cleaning supplies referral rates. The results show a smaller increase in referral rates for rental assistance. For uptake, we observe a 3.26% (1.47%-5.06%) in the overall uptake rate and a 2.08% (0.93%-3.22%) increase in the rental assistance, a 2.46% (0.79%-4.14%) increase in the food assistance, and a 2.48% (0.81%-4.15%) increase in the cleaning supplies uptake rates. We do not find evidence that the program affected the motel placement referral or uptake rate.

**Table 2. Effect of the high-touch program on referral rates and uptake rates related to Isolation & Quarantine Support Program (IQSP) services as estimated by a difference-in-differences (DiD) analysis.**

|  |  | Effect | SE | 95% CI | $p$-value |
| --- | --- | --- | --- | --- | --- |
| **Referral rate (%)** | Overall | 4.18** | 1.62 | [ 1.00, 7.37] | 0.01 |
|  | Cash assistance | 4.00*** | 0.89 | [ 2.26, 5.75] | <0.01 |
|  | Rental assistance | 0.81** | 0.32 | [ 0.18, 1.44] | 0.01 |
|  | Food assistance | 2.78** | 1.09 | [ 0.63, 4.92] | 0.01 |
|  | Cleaning supplies | 2.01*** | 0.63 | [ 0.77, 3.25] | <0.01 |
|  | Motel placement | 0.42 | 0.38 | [-0.32, 1.17] | 0.27 |
| **Uptake rate (%)** | Overall | 3.26*** | 0.92 | [ 1.47, 5.06] | <0.01 |
|  | Rental assistance | 2.08*** | 0.58 | [ 0.93, 3.22] | <0.01 |
|  | Food assistance | 2.46*** | 0.85 | [ 0.79, 4.14] | <0.01 |
|  | Cleaning supplies | 2.48*** | 0.85 | [ 0.81, 4.15] | <0.01 |
|  | Motel placement | -0.02 | 0.03 | [-0.08, 0.05] | 0.61 |

Author’s analysis of CalCONNECT and IQSP data. Effects, standard errors, and 95% confidence interval values are reported as percentages. The data for this evaluation consists of 79,123 cases for the DiD analysis. SE = standard error. $***p<0.01, **p<0.05, *p<0.10$.

To test the validity of our pre-treatment period range selection, we conduct a placebo test. In Fig 2, we fit a DiD on the first two periods representing the placebo pre-treatment period and the third time period representing the placebo post-treatment period. The y-axis represents the estimated effect of the placebo treatment on the outcome (here, the overall referral rate). The dots represent estimates for each fitted model, with the vertical lines representing 95% confidence intervals. The left panel shows the estimates for time periods excluding any dates from the true model on a moving window of three time periods at a time, with the treatment period (January 6, 2021 and onwards) while the right panel includes those dates (colored in green).

**Fig 2.** **Placebo test of differences-in-differences (DiD) analysis using moving windows of three time periods.**


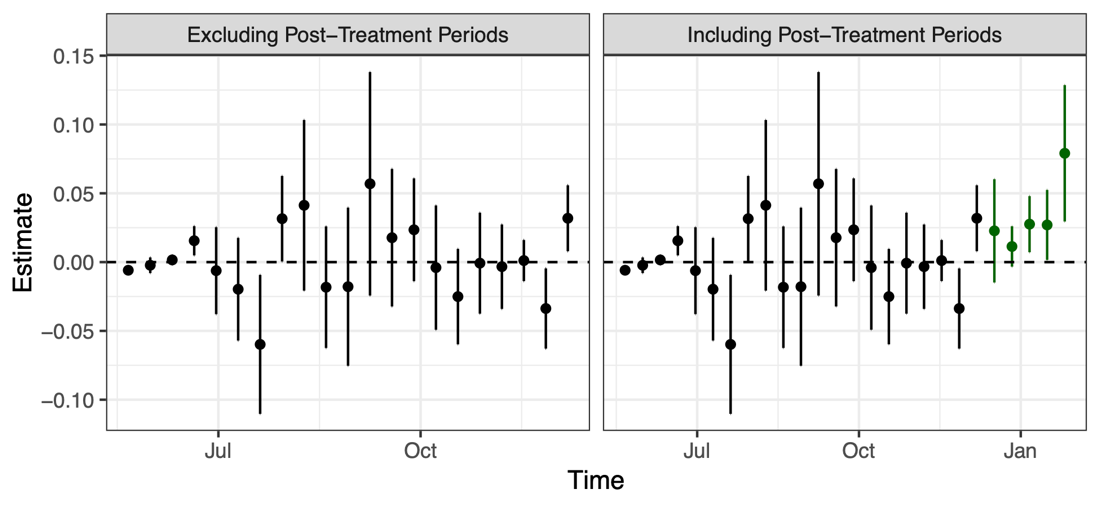


Author’s analysis of CalCONNECT data. The y-axis presents the DiD estimate and the x-axis represents time. Each window contains three time periods, with a time period being 10 days. The first two time periods are taken to be the placebo pre-treatment period and the third time period is the placebo post-treatment period. Each dot represents the placebo effect of the program on referral rate as estimated by a DiD model fitted on the three time periods of data. The vertical lines represent the 95% confidence intervals. The left panel depicts the estimates excluding data from the actual program, while the right panel includes that data (with estimates modeled on that data colored in green).

In the left panel, while most estimates contain the origin, there is some noise around the time of the second wave of the pandemic, leading to false rejections around July and August. The right panel shows the upward trend of estimates as more data from the true treatment period is included, corroborating the treatment effect of the intervention. This moving window analysis is consistent with our decision to trim the DiD pre-treatment period and include only data after November 7, 2020 for the analysis.

1. Agency for Toxic Substances and Disease Registry. CDC/ATSDR Social Vulnerability Index [Internet]. Place and Health. 2022. Available from: https://www.atsdr.cdc.gov/placeandhealth/svi/index.html

2. Santa Clara County Public Health Department. Social Vulnerability Index [Internet]. County of Santa Clara Open Data Portal. 2022. Available from: https://data-sccphd.opendata.arcgis.com/maps/sccphd::social-vulnerability-index-1/explore

3. County Opening New High-Capacity Testing Site at Fairgrounds Testing 1,000 People Daily by Friday and Up To 5,000 in the Future - Emergency Operations Center - County of Santa Clara [Internet]. [cited 2022 Apr 21]. Available from: https://covid19.sccgov.org/news-releases/pr-08-18-2020-new-high-capacity-testing-site-at-fairgrounds

4. COVID-19 Testing Aug. 10 to Aug. 14: Sites in San Jose, Gilroy, Sunnyvale, Morgan Hill, Santa Clara, Los Altos, Palo Alto - Emergency Operations Center - County of Santa Clara [Internet]. [cited 2022 Apr 21]. Available from: https://covid19.sccgov.org/news-releases/pr-08-08-2020-covid-19-testing-august-10-14
