# Supplementary material for: Integrating social services with disease investigation: A randomized trial of COVID-19 high-touch contact tracing
Source: PLoS One. 2023 May 16;18(5):e0285752. doi: 10.1371/journal.pone.0285752 (PMC10187910; doi:10.1371/journal.pone.0285752)
Supplement: S3 Appendix — (DOCX) [file pone.0285752.s003.docx]

## S3 Appendix. Cluster Randomized Trial.

To estimate the intention-to-treat (ITT) effects, we use the following regression:

$$Y_{i}=\alpha+\delta Z_{i}+\varepsilon_{i}$$

$Y_{i}$ represents the outcome. $Z_{i}$ is a dummy variable indicating the random (ITT) assignment of client $i$. $\varepsilon_{i}$ is an error term. $\alpha$ is an intercept. $\delta$ is a regression coefficient.

To estimate the causal average complier effects, we use the following regressions:

$$Y_{i}=\alpha+\delta D_{i}+\rho X_{i}+\varepsilon_{i}$$

$$D_{i}=\gamma+\beta Z_{i}+\pi X_{i}+\epsilon_{i}$$

$Y_{i}$ represents the outcome. $Z_{i}$ is a dummy variable indicating the random (ITT) assignment of client $i$. $D_{i}$ is a dummy variable indicating if client $i$ actually received treatment, *i.e.*, it equals 1 if the client was actually assigned to a high-touch contact tracer and 0 if the client was actually assigned to a standard contact tracer. $X_{i}$ represents demographic and time controls. $\varepsilon_{i}$ and $\epsilon_{i}$ are error terms. $\alpha$ and $\gamma$ are intercepts. $\delta$, $\rho$, $\beta$, and $\pi$ are regression coefficients. We estimate the complier average causal effects (CACE) with the *iv_robust* function from the estimatr R package, which implements a two-stage least squares IV estimator [1].

Fig 1 illustrates the randomization protocol used during the week of March 29, 2021. Each dot in the figure represents a ZIP Code in Santa Clara County, plotted by the weekly positivity rate and SVI. For that particular week, the SVI threshold was 49.12 and the positivity rate threshold was 1.45%. The dot size corresponds to the previous week’s case count in that ZIP Code.

**Fig 1. Scatter plot of Santa Clara County ZIP Codes by social vulnerability index (SVI) and positivity rate for the week of March 29, 2021.**


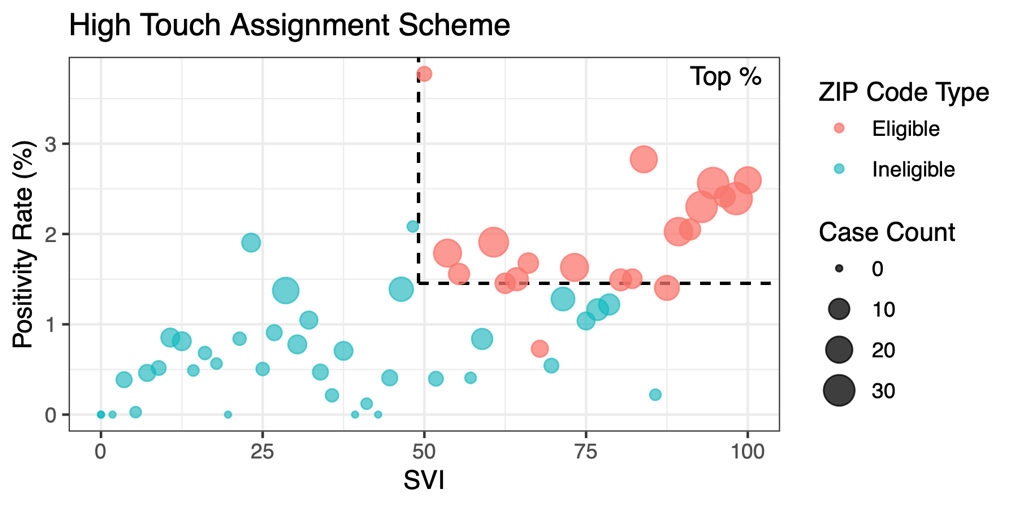


Author’s analysis of CalCONNECT data. Each dot on the scatter plot represents a ZIP Code in Santa Clara County, with the size corresponding to that ZIP Code’s case volume in the previous week. Each week, ZIP Codes were determined to be eligible for assignment to the high-touch team if they fell in the top percentile of SVI and weekly positivity rate, shown here as the upper right quadrant defined by the dashed lines. Of the eligible ZIP Codes, half were then randomly assigned to the high-touch team (treatment) and half were routed through the normal contact tracing protocol (control). Certain vulnerable ZIP Codes were deemed always eligible (e.g., the red dot outside of the quadrant), but these often coincided with the eligible quadrant. The top percentile was initially set by operational capacity and later set to the upper half of SVI and upper third of weekly positivity rates.

There were two types of deviations from randomization protocol required by the County per health equity objectives. First, in specific weeks at the start and end of the trial, priority ZIP Codes from the original launch were deemed critical to support with high-touch services, and were thus non-randomly assigned to the high-touch team. On the week of February 15, 2021, ZIP Codes 95116, 95122, and 95127 were non-randomly assigned. From the week of April 12 through the week of May 17, 2021, ZIP Codes 95111, 95112, 95116, 95121, 95122, 95127, 95133, and 95148 were non-randomly assigned. 1,110 cases were subject to this non-random assignment and excluded from the main analysis. As an additional exception to the scheme, cases (fewer than 50) routed from two other community interventions, door-to-door testing in East San Jose and a partnership with a local community-based health center, would always be routed to high-touch contact tracers, as giving clients access to the high-touch program was part of that intervention [2]. Any such cases that were not already excluded remained in the analysis.

Fig 2 illustrates the degree of noncompliance present in both the treatment and control groups. The top panel shows all 8,216 cases in Santa Clara County eligible during the study period, including the 1,110 cases in ZIP Codes that were non-randomly assigned, presented here for purposes of understanding overall compliance. The bottom panel shows only the 5,430 cases that were randomized and used in the main analysis. The disruptions in mid-April, consistent with the deviations from protocol, reflect the need to account for noncompliance using an IV analysis.

**Fig 2. Compliance, or probability of treatment, for the treatment (in red) and control groups (in blue) by week over the randomized expansion period.**


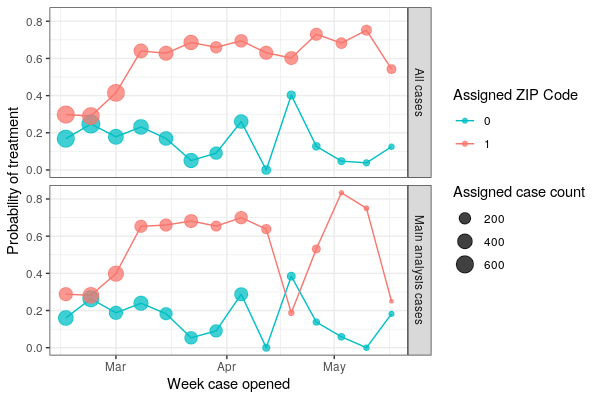


Author’s analysis of CalCONNECT data. The top panel plots all 8,216 eligible cases in Santa Clara County during the study period. The bottom panel plots 5,430 randomized cases used in the main analysis. The size of the dot corresponds to the weekly case count.

Since we exclude ZIP Codes that did not opt into all of the County’s IQSP services in both the ITT and IV analyses, we recalculate both analyses as well as the balance check with all ZIP Codes (thereby considering all 7,106 randomized cases in Santa Clara County) as a sensitivity check. The resultant dataset consists of 3,415 cases in the treatment group and 3,691 cases in the control group. Table 1 provides recalculated covariate balance statistics on all 7,106 randomized cases, covering all ZIP Codes in Santa Clara County. We observe that balance is still maintained and that there is still a strong instrument (F- statistic is 181.01). Table 2 reports the recalculated results of the IV analysis including all 7,106 cases. The results are similar, though attenuated for many of the outcomes.

**Table 1. Balance check on individual-level demographic attributes between treatment group (cases randomly assigned to a high-touch contact tracer) and control group (cases not assigned to a high-touch contact tracer), including all cases in Santa Clara County as a sensitivity check.**

|  |  | Control | Treatment | 95% CI | $p$-value |
| --- | --- | --- | --- | --- | --- |
| Gender | Male | 0.53 | 0.51 | [-0.01, 0.04] | 0.28 |
|  | Female | 0.47 | 0.48 | [-0.03, 0.01] | 0.34 |
|  | Unknown/other | 0.01 | 0.01 | [-0.01, 0.00] | 0.49 |
| Race/ethnicity | Latinx | 0.47 | 0.47 | [-0.03, 0.02] | 0.90 |
|  | White | 0.14 | 0.14 | [-0.02, 0.01] | 0.73 |
|  | Asian | 0.23 | 0.23 | [-0.02, 0.02] | 0.88 |
|  | Black | 0.02 | 0.02 | [-0.01, 0.01] | 0.90 |
|  | Not reported | 0.05 | 0.05 | [-0.01, 0.01] | 0.47 |
|  | Multirace/other | 0.08 | 0.07 | [-0.01, 0.02] | 0.34 |
| Age (y) | Mean age | 36.34 | 36.10 | [-0.64, 1.13] | 0.59 |
|  | 0-17 | 0.17 | 0.17 | [-0.02, 0.02] | 0.79 |
|  | 18-24 | 0.13 | 0.13 | [-0.02, 0.01] | 0.31 |
|  | 25-44 | 0.38 | 0.37 | [-0.02, 0.03] | 0.56 |
|  | 45-64 | 0.25 | 0.26 | [-0.02, 0.02] | 0.73 |
|  | 65+ | 0.07 | 0.07 | [-0.01, 0.01] | 0.67 |
| Sample size |  | 3,691 | 3,415 |  |  |

Author’s analysis of CalCONNECT data.

**Table 2. Effect of the high-touch program on referral rates and uptake rates related to Isolation & Quarantine Support Program (IQSP) services as estimated by an intention-to-treat (ITT) analysis on the left-hand side and an instrumental variables (IV) analysis on the right-hand side, including all cases in Santa Clara County as a sensitivity check.**

|  |  | **Intention-to-treat** | | | | **Instrumental variables** | | | |
| --- | --- | --- | --- | --- | --- | --- | --- | --- | --- |
|  |  | Effect | SE | 95% CI | $p$-value | Effect | SE | 95% CI | $p$-value |
| **Referral rate (%)** | Overall | 2.26* | 1.16 | [-0.01, 4.54] | 0.05 | 6.47** | 3.26 | [ 0.04, 12.90] | 0.05 |
|  | Food assistance | 1.63** | 0.83 | [ 0.01, 3.25] | 0.05 | 4.86** | 2.32 | [ 0.28, 9.44] | 0.04 |
|  | Cash assistance | 0.77 | 0.71 | [-0.63, 2.17] | 0.28 | 2.06 | 2.06 | [-2.00, 6.12] | 0.32 |
|  | Cleaning supplies | 0.37 | 0.60 | [-0.81, 1.56] | 0.54 | 1.00 | 1.74 | [-2.44, 4.43] | 0.57 |
|  | Motel placement | -0.33 | 0.29 | [-0.89, 0.23] | 0.25 | -1.05 | 0.89 | [-2.81, 0.71] | 0.24 |
|  | Rental assistance | -0.78 | 0.55 | [-1.85, 0.29] | 0.15 | -2.42 | 1.57 | [-5.52, 0.68] | 0.13 |
|  |  |  |  |  |  |  |  |  |  |
| **Uptake rate (%)** | Overall | 1.22 | 0.94 | [-0.62, 3.06] | 0.19 | 3.55 | 2.68 | [-1.73, 8.83] | 0.19 |
|  | Food assistance | 1.20 | 0.74 | [-0.25, 2.64] | 0.11 | 3.65* | 2.12 | [-0.53, 7.83] | 0.09 |
|  | Cleaning supplies | 1.11 | 0.73 | [-0.33, 2.54] | 0.13 | 3.35 | 2.09 | [ 0.78, 7.48] | 0.11 |
|  | Rental assistance | 0.43 | 0.69 | [-0.92, 1.79] | 0.53 | 1.20 | 2.00 | [-2.75, 5.15] | 0.55 |
|  | Motel placement | 0.07 | 0.11 | [-0.15, 0.28] | 0.55 | 0.20 | 0.34 | [-0.46, 0.86] | 0.56 |

Author’s analysis of CalCONNECT and IQSP data. Effects, standard errors, and 95% confidence interval values are reported as percentages. The data for these evaluations consists of 7,106 cases. SE = standard error. $**p<0.05, *p<0.10$.

Table 3 provides an alternative version of the ITT analysis on the main study’s 5,430 cases, performed as a logistic regression with demographic covariates (gender, race/ethnicity, age), time (week) fixed effects, and clustered standard errors by week-ZIP Code. The effect reported is the odds ratio of the outcome, between cases in the high-touch program and cases in standard contact tracing.

**Table 3. Odds ratio of referral rates and uptake rates related to Isolation & Quarantine Support Program (IQSP) services, between cases in the high-touch program and cases in standard contact tracing, as estimated by an intention-to-treat (ITT) analysis.**

|  |  | **Intention-to-treat** | | | |
| --- | --- | --- | --- | --- | --- |
|  |  | Effect | SE | 95% CI | $p$-value |
| **Referral rate (%)** | Overall | 1.0265** | 1.0126 | [1.0016, 1.0520] | 0.04 |
|  | Food assistance | 1.0209** | 1.0087 | [1.0035, 1.0385] | 0.02 |
|  | Cash assistance | 1.0128* | 1.0075 | [0.9981, 1.0277] | 0.09 |
|  | Cleaning supplies | 1.0048 | 1.0067 | [0.9918, 1.0181] | 0.47 |
|  | Motel placement | 0.9953 | 1.0034 | [0.9886, 1.0020] | 0.17 |
|  | Rental assistance | 0.9931 | 1.0059 | [0.9818, 1.0046] | 0.24 |
|  |  |  |  |  |  |
| **Uptake rate (%)** | Overall | 1.0153* | 1.0084 | [0.9987, 1.0322] | 0.07 |
|  | Food assistance | 1.0154** | 1.0070 | [1.0016, 1.0294] | 0.03 |
|  | Cleaning supplies | 1.0148** | 1.0071 | [1.0009, 1.0290] | 0.04 |
|  | Rental assistance | 1.0053 | 1.0071 | [0.9915, 1.0193] | 0.45 |
|  | Motel placement | 1.0000 | 1.0014 | [0.9972, 1.0028] | 0.98 |

Author’s analysis of CalCONNECT and IQSP data. Effects, standard errors, and 95% confidence interval values are reported as odds ratios. The data for these evaluations consists of 5,430 cases. SE = standard error. $**p<0.05, *p<0.10$.

1. Graeme Blair, Jasper Cooper, Alexander Coppock, Macartan Humphreys, Luke Sonnet. estimatr: Fast Estimators for Design-Based Inference [Internet]. 2022 [cited 2022 Jun 20]. Available from: https://declaredesign.org/r/estimatr/, https://github.com/DeclareDesign/estimatr

2. Chugg B, Lu L, Ouyang D, Anderson B, Ha R, D’Agostino A, et al. Evaluation of allocation schemes of COVID-19 testing resources in a community-based door-to-door testing program. In: JAMA Health Forum. American Medical Association; 2021. p. e212260–e212260.
