# Supplementary material for: Integrating social services with disease investigation: A randomized trial of COVID-19 high-touch contact tracing
Source: PLoS One. 2023 May 16;18(5):e0285752. doi: 10.1371/journal.pone.0285752 (PMC10187910; doi:10.1371/journal.pone.0285752)
Supplement: S4 Appendix — (DOCX) [file pone.0285752.s004.docx]

## S4 Appendix. Survey.

We administered a survey to all active high-touch contact tracers in November 2021 with the intent of understanding how the program affected health outcomes not measurable from CalCONNECT data, such as physical health, mental health, or other social determinants of health. Table 1 lists the questions fielded to the tracers. 18 of the 26 active tracers at the time responded (69.23% response rate). Fig 1 summarizes the results to the first two questions, which ask respondents to estimate the distribution of their clients’ risk and engagement levels. There is no discernible difference in the proportions of risk, but most respondents report clients being moderately or highly engaged. Table 2 presents a sample of responses to the remaining free-form questions in the survey representing the themes that emerged from the answers: improved client mental health; greater client sense of stability from having social determinants of health addressed; increased client knowledge and awareness of resources; improved physical health outcomes for clients with comorbidities; and improved contact tracer morale.

**Table 1. Summary of survey questions fielded to active high-touch contact tracers in November 2021.**

| **High-Touch Contact Tracing Survey Questions**  Client Population/Perspective   1. Please estimate the percentage of high-touch clients you encountered for each level of risk. The sum of all groups should equal 100.    1. No high-touch services: Client declines resources; is confident navigating resources on their own. _______    2. Low risk: Client is followed up with on day 7; is comfortable navigating resources on their own. _______    3. Medium risk: Client is followed up with on day 2-4; is not very familiar with resource navigation and/or may have multiple stressors preventing them from reaching out to resources themselves. _______    4. High risk: Client is followed up with on day 1-2; is uncomfortable and/or unable to navigate resources on their own. _______ 2. Please estimate the percentage of high-touch clients you encountered by level of engagement or trust towards the high-touch program. The sum of all groups should equal 100.    1. Little to no engagement: Client is distrustful or way, may try to end the conversation as quickly as possible. _______    2. Moderate engagement: Client is somewhat open to receiving assistance but may be less forthcoming about providing information or contacts. _______    3. High engagement: Client is comfortable with the program and providing details about their personal life and/or contacts during interviews. _______   Health Outcomes   1. What is your sense of how these services have affected the physical health outcomes of clients? Please provide examples, if available. 2. What is your sense of how these services have affected other outcomes? Please feel free to comment on mental health, childcare, housing stability, food access, and other social determinants of health.   Other   1. Please offer any other comments or reflections on your experience conducting high-touch contact tracing here. |
| --- |

Respondents were asked to estimate the client population’s distribution of risk level (defined in Table 1 in S1 Appendix) and engagement. Respondents were also given the opportunity to elaborate on outcomes not captured in the measurable outcomes, *e.g.*, physical health outcomes and other social determinants of health.

**Fig 1.** **Distribution of risk and engagement level across the high-touch client population as estimated by survey respondents.**


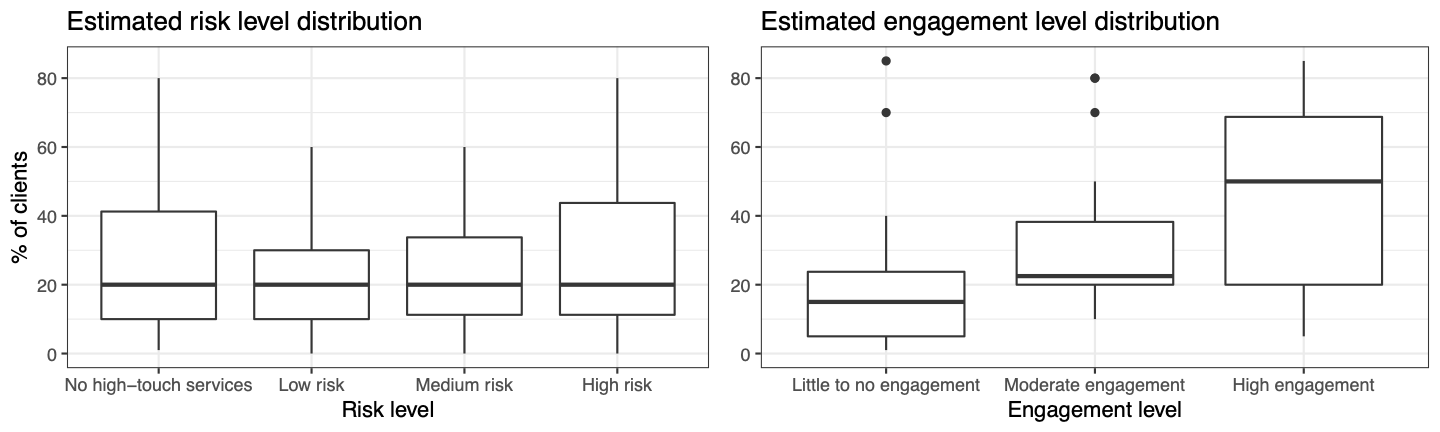


Author’s analysis of survey data. The y-axis represents the percentage of high-touch clients served by the respondent falling into the risk or engagement level on the x-axis.

**Table 2.** **Sample of responses to survey of high-touch contact tracers illustrating perceptions on how the program impacted client health outcomes and contact tracer morale.**

| **Sample of High-Touch Contact Tracing Survey Responses**  “[The program] provided knowledge not only to an individual but to an entire household and entire communities…often times a simple follow up call in a few days was very therapeutic in the sense that having somebody check up on a case while isolating liberated [them from] possible boredom and lonely feelings. In extreme circumstances such as homelessness, hotel placement was a blessing in order to properly isolate and be able to rest in a safe environment to ultimately recover from [COVID-19]. In other cases food and financial [assistance] relieve[d] stress and provided [a] sense of stability.”  “Overall, this high[-]touch program not only has helped the community but it has also helped me as a contact tracer. In my case, it has brought a sense of accomplishment, purpose, and it allows me to indulge in what I love most…This pandemic affected my own family from the early stages tremendously. Looking back it was such a scary and emotional situation that brought a lot of confusion. Because of my own experiences with the pandemic, being a part of CICT and [the high-touch] program is very rewarding. Being able to help a family who felt how my family and I did in the past is what makes all the hard work worth it.”  “Our calls do a massive amount of the mental health lifting for these people, having someone checking on them, someone to vent to and someone who cares on the other side of the line, at the tip of you[r] fingers and a phone call…helping [troubleshoot] and carry the aggregate mental load that this causes people.”  “I believe these services helped tremendously in the physical health outcomes of the cases I handled. Had a case who had a heart condition and was diabetic. I was providing close [monitoring and support] and I ended up calling her son on her behalf to inform him his mother needed medical attention since her sugar level had dropped very low. I also provided a clinical referral who provided instructions to the son on how to get sugar level back up for case and taking her to her PCP.”  “Other outcomes that I've seen [have] been food access and housing stability through our [IQ] services—clients are often grateful when we are able to provide them food—especially those who are unable to purchase food due to income and having to isolate. Others, who are homeless and/or in crowded households have benefitted from motel/shelter placements.” |
| --- |

Author’s analysis of survey data. CICT = Case Investigation and Contact Tracing Team, PCP = primary care provider. N=18.
